# Effects of TBBPA Exposure on Neurodevelopment and Behavior in Mice

**DOI:** 10.3390/ijms26157289

**Published:** 2025-07-28

**Authors:** Yongin Kim, Inho Hwang, Sun Kim, Eui-Bae Jeung

**Affiliations:** Laboratory of Veterinary Biochemistry and Molecular Biology, College of Veterinary Medicine, Chungbuk National University, Cheongju 28644, Republic of Korea; scott7852@gmail.com (Y.K.); darkpower777@nate.com (I.H.); boksagi88@gmail.com (S.K.)

**Keywords:** Tetrabromobisphenol A, endocrine disrupting chemicals, developmental neurotoxicity, neurodevelopment, behavioral disorders

## Abstract

Tetrabromobisphenol A (TBBPA) is a brominated flame retardant widely used in consumer products. TBBPA is often detected in soil, water, organisms, and even in human blood and breast milk. Hence, it is accessible to developing fetuses and nursing offspring after maternal exposure. The reported evidence for the endocrine disruption of TBBPA in the brain has raised concerns regarding its effects on neurodevelopmental and behavioral functions. This study investigated the effects of TBBPA exposure on neurodevelopment. A cell-based developmental neurotoxicity assay was performed to determine whether TBBPA is a developmental neurotoxicant. The assay revealed TBBPA to be a developmental neurotoxicant. C57BL/6N maternal mice were administered TBBPA at 0, 0.24, and 2.4 mg/kg during pregnancy and lactation, and their offspring underwent behavioral testing. The behavioral experiments revealed sex-specific effects. In females, only a deterioration of the motor ability was observed. In contrast, deteriorations in motor function, memory, and social interaction were noted in males. Furthermore, we validated changes in the expression of genes associated with behavioral abnormalities, confirming that perinatal exposure to TBBPA, at the administered doses, can affect neurodevelopment and behavior in offspring. These findings highlight the need for more in-depth and multifaceted research on the toxicity of TBBPA.

## 1. Introduction

Tetrabromobisphenol A (TBBPA) accounts for 60% of the total brominated flame retardant (BFR) market [1], and the global production of TBBPA and its derivatives is estimated to be more than 100,000 tons [2]. A previous study reported the annual consumption of TBBPA to be 210,000 tons [3]. TBBPA is used primarily as a reactive type of flame retardant in polycarbonate and epoxy resins for circuit boards, and is employed in more than 70% of electronic products worldwide [4]. Only approximately 10% of TBBPA is used as a reactive type in several polymers [5]. When used as an additive ingredient, TBBPA is not part of the polymer constitution and can be released easily. On the other hand, when used as a reactive component, TBBPA becomes incorporated into the polymer structure, making it difficult to release [6]. Despite the characteristics of reactive TBBPA, the additive and reactive forms have been shown to release TBBPA and its metabolites into the environment [7,8]. Consequently, TBBPA has been detected in air, water, soil, and dust [9,10].

TBBPA is prone to bioaccumulation in humans and other animals via the food chain. Morris et al. (2004) examined the accumulation of TBBPA in the body of aquatic organisms in the North Sea [11]. They detected up to 245 ng/g fat of TBBPA in whiting and up to 418 ng/g fat in harbor porpoises. Shi et al. (2009) reported that the average concentration of TBBPA was 0.263 ng/g fat, 0.194 ng/g fat, 0.738 ng/g fat, and 0.211 ng/g fat in meat, eggs, seafood, and milk, respectively [12]. TBBPA is frequently found in human tissues, serum, and milk [13,14,15]. The data from a Chinese study revealed up to 5.134 ng/g fat TBBPA concentrations in breast milk [13]. According to a monitoring study conducted in France, the maximum TBBPA concentration of samples from women was 37.34 ng/g fat and 649.45 ng/g fat in breast milk and cord serum, respectively [15]. Hence, fetal exposure can occur through the placenta and exposure in infancy through breastfeeding. These frequent detections have raised concerns about the potential effects of TBBPA.

Initially, TBBPA was widely used, as it was known to be a non-toxic substance. The European chemicals bureau (ECB) and the world health organization (WHO) reported that exposure to TBBPA is unlikely to have a negative impact on consumers [16,17]. However, as research on TBBPA progressed, various toxic effects began to be reported. The international agency for research on cancer (IARC) recently classified TBBPA as group 2A, probably carcinogenic to humans, based on the evidence for carcinogenicity in laboratory animals [2]. Several researchers have reported that TBBPA can cause endocrine disruption in androgen, estrogen, and thyroid signaling, and reproductive toxicity with potential effects on brain development. Therefore, this chemical requires in-depth evaluation for multiple toxicities [18].

TBBPA is similar in structure to estrogen and thyroxine (T4), and is considered an endocrine-disrupting chemical (EDC) [19,20]. TBBPA disrupts estradiol sulfurylation in vitro [21]. TBBPA induced cell death in the testes and abnormal expression of testicular genes in CD1 mice [22]. Sanders et al. (2016) provided evidence that estrogen signaling, biosynthesis, and metabolism were altered in the uterus and liver of rats exposed to TBBPA [23]. A negative correlation was observed between TBBPA and triiodothyronine (T3) [24]. TBBPA exposure revealed a significant adverse effect on the sperm count, and reduced the content of thyroid-stimulating hormone (TSH), T3, and T4 [25]. Sex steroids are important for numerous aspects of brain organization, particularly sexual differentiation [26,27]. Furthermore, a disruption of thyroid hormone signaling leads to detrimental neurodevelopmental outcomes, such as cognitive impairment [28]. These data provide the basis to infer that TBBPA exposure can exert influence on neurodevelopment.

TBBPA, a type of EDC, is increasingly being reported as a potential neurodevelopmental toxicant. Studies of the developmental neurotoxicity of TBBPA in rodent models have shown that TBBPA can affect auditory response, motor ability, learning, and memory [29,30,31]. Parental exposure to TBBPA during mating, pregnancy, and lactation affected the auditory responses of offspring in Wistar rats [29]. The acute exposure of male mice to TBBPA increased memory slightly and altered horizontal movement activity [30]. The subacute treatment of mice administered TBBPA from 42 to 56 days of life induced memory aggravation in a passive avoidance test [31]. The administration of TBBPA to pregnant rats caused an increase in anxiety in male pups [32].

Previous studies reported that acute, subacute, and developmental TBBPA exposure in rodents has adverse effects on several types of behavior. Notably, a few studies, such as one in Wistar rats [32], have reported sex-specific behavioral changes following TBBPA exposure. However, many of these studies either focused on limited behavioral domains, administered very high doses, or employed extended postnatal exposure schedules beyond early development. There is a paucity of research data regarding (1) the behavioral effects of developmental exposure at subchronic, non-extreme doses, (2) the effects according to gender, and (3) more comprehensive impact analysis through various behavioral experiments. Moreover, few studies have integrated behavioral findings with gene expression analyses to investigate potential mechanisms. Therefore, this study examined the effects of exposure to TBBPA with the hypothesis that adverse effects would appear in one or both genders in several behavioral experiments, and that these effects would be accompanied by sex-dependent changes in neurobehavioral gene expression.

## 2. Results

### 2.1. TBBPA Is a Developmental Neurotoxicant

**Figure 1:**
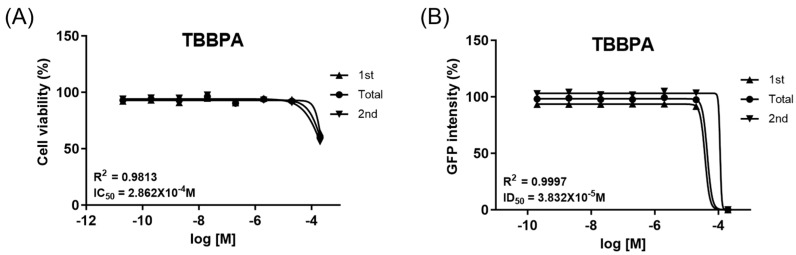
Determination of the developmental neurotoxicity of TBBPA through a Sox1-GFP-based cell screening assay. (**A**) Results of cell viability test, *n* = 6 for each concentration. The coefficient of determination, *R*^2^, was 0.9813. (**B**) GFP intensity from neuronal differentiation test, *n* = 8 for each concentration. The *R*^2^ value was 0.9997. No statistical tests were performed.

The adverse effects of TBBPA on neurodevelopment were predicted using a Sox1-GFP-based screening assay with mouse embryonic stem cells. This test had two endpoints. The first endpoint was the IC_50_ value of Sox1-GFP cells, which was assessed using a CCK-8 assay (Figure 1A). The second endpoint, ID_50_ of TBBPA on Sox1-GFP mESC, was evaluated (Figure 1B). The IC_50_ and ID_50_ are the half-maximal inhibitory concentration of TBBPA and the half-maximal inhibitory differentiation dose for the neural differentiation of the cells, respectively, and were determined to be 2.862 × 10^−4^ M and 3.832 × 10^−5^ M, respectively. The values were substituted into the discriminant function reported by Park et al. (2021) and validated by Jeong et al. (2022) [33,34]. The discriminant function score was −0.75003, meaning that TBBPA is developmental-neurotoxic-positive.

### 2.2. Maternal Exposure to TBBPA Occured During Pregnancy and Lactaiton

#### 2.2.1. Maternal Exposure to TBBPA Impaired Locomotor Ability but Not Anxiety

**Figure 2:**
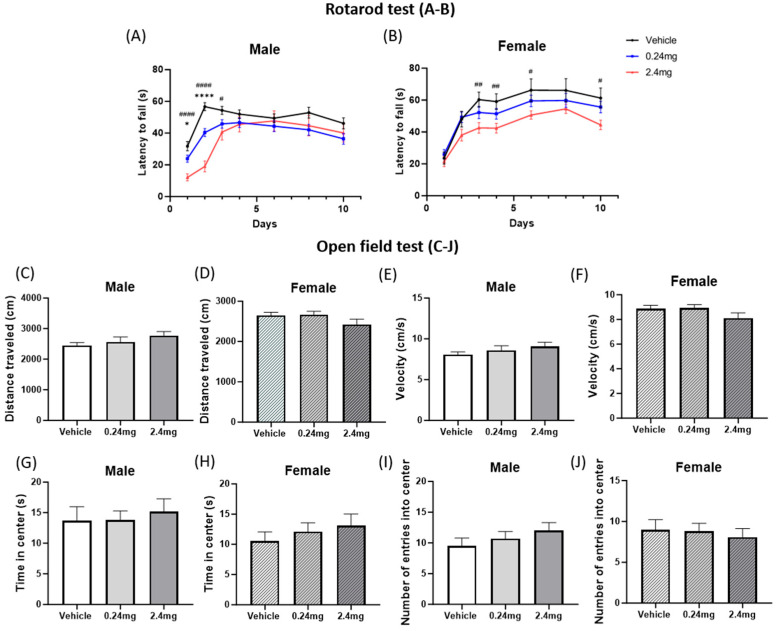
Effects of TBBPA on locomotor activity and anxiety-like behavior. (**A**,**B**) Rotarod test results. *n* = 15 males/11 females for vehicle, 19 males/16 females for TBBPA 0.24 mg/kg, 7 males/13 females for TBBPA 2.4 mg/kg. (**C**–**J**) Open-field test results: (**C**,**D**) distance moved; (**E**,**F**) velocity; (**G**,**H**) time spent in the center; (**I**,**J**) number of entries into the center. Open-field test groups used the same n as that in the rotarod test. The data are presented as the means ± SEM. Statistical significance was determined by a one-way ANOVA with a Dunnett’s multiple comparisons test. Data were analyzed per individual animal and not on a litter basis. * *p* < 0.05 vehicle vs. TBBPA 0.24 mg/kg, **** *p* < 0.0001 vehicle vs. TBBPA 0.24 mg/kg, # *p* < 0.05 vehicle vs. TBBPA 2.4 mg/kg, ## *p* < 0.01 vehicle vs. TBBPA 2.4 mg/kg, #### *p* < 0.0001 vehicle vs. TBBPA 2.4 mg/kg.

The mice offspring were subjected to behavioral testing to assess the effects of exposure to TBBPA on murine brain functioning. The locomotor activity of the mice was estimated using a rotarod test. For males, the latency to fall significantly decreased compared to the vehicle group on day 1 (*p* = 0.0418) and day 2 (*p* < 0.0001) in the 0.24 mg/kg group, and on day 1 (*p* < 0.0001), day 2 (*p* < 0.001), and day 3 (*p* = 0.0162) in the 2.4 mg/kg group (Figure 2A). On the other days, there was no significance throughout the training and test phases. For females, the group exposed to 2.4 mg/kg of TBBPA consistently showed reduced motor learning ability with significant decrease of the latency to fall on day 3 (*p* = 0.006), day 4 (*p* = 0.0061), day 6 (*p* = 0.0371), and day 10 (*p* = 0.0166) compared to the vehicle group (Figure 2B). The open field test was conducted to evaluate the motor ability and anxiety. The distance traveled and velocity, which reflect the motor ability, were measured, but there was no significant difference between the three groups for males and females (Figure 2C–F). The time in the center and the number of entries into the center, which reflect anxiety, also showed no significant differences (Figure 2G–J). These results suggest that perinatal exposure to TBBPA can partially impair the motor function but not affect anxiety.

#### 2.2.2. Exposure to TBBPA Did Not Affect Depression-Related Behavior

**Figure 3:**
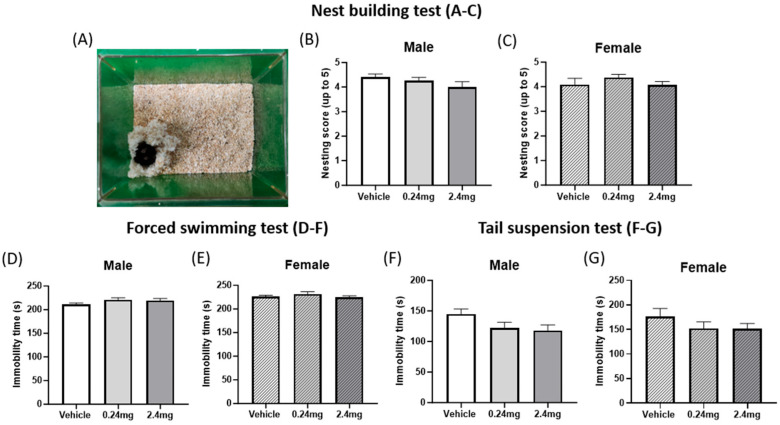
Effects of TBBPA on depression-related behaviors. (**A**) Representative image of the nest building test. (**B**,**C**) Nest building test scores. *n* = 15 males/11 females for vehicle, 19 males/16 females for TBBPA 0.24 mg/kg, 7 males/13 females for TBBPA 2.4 mg/kg. (**D**,**E**) Immobile time in the forced swimming test (FS). (**F**,**G**) Immobile time in the tail suspension test (TS). FS and TS groups used the same n as in the nest building test. The data are plotted as the means ± SEM. The statistical analysis was determined by a one-way ANOVA with a Dunnett’s multiple comparisons test. Data were analyzed per individual animal and not on a litter basis.

The nest building test, forced swimming test, and tail suspension test were performed to determine how TBBPA affects depression-related behavior in mice. The test results showed no significant difference between the vehicle and administration groups in the nest building ability (Figure 3A–C). Likewise, the immobility time in the forced swimming and tail suspension tests to assess depressive behavior, and the results also indicated no significant difference between the experimental and control groups (Figure 3D–G), suggesting that TBBPA does not affect depression-related behaviors in mouse offspring.

#### 2.2.3. Exposure to TBBPA Led to a Deterioration in Spatial Learning and Memory

**Figure 4:**
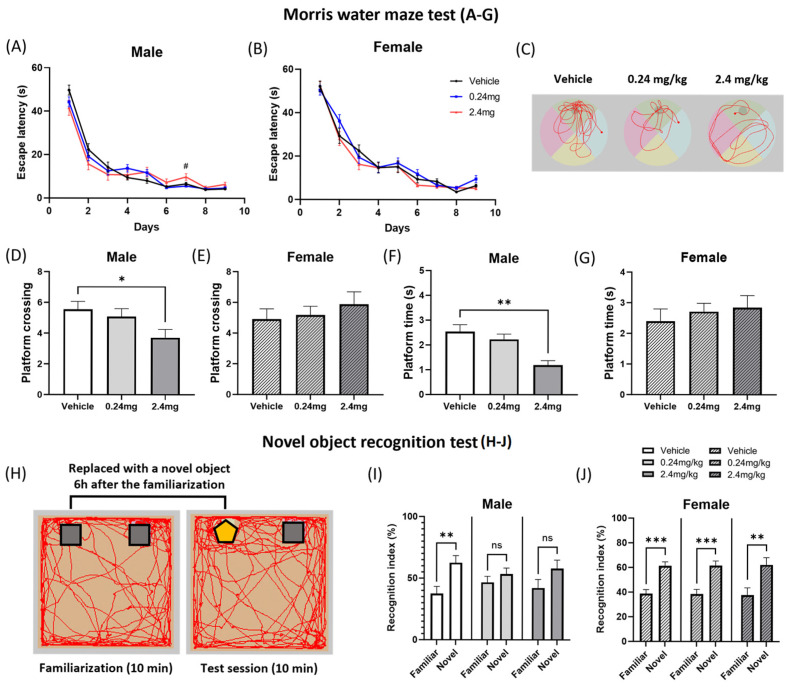
Effects of TBBPA on learning and memory in offspring mice. (**A**–**G**) Morris water maze test (MWM) results: *n* = 15 males/11 females for vehicle, 19 males/16 females for TBBPA 0.24 mg/kg, 7 males/13 females for TBBPA 2.4 mg/kg. (**A**,**B**) Escape latency of mice for nine consecutive training periods. (**C**) Representative image of mouse movement tracking in the test session. (**D**,**E**) Frequency of crossing the platform. (**F**,**G**) Time spent in the platform area. (**H**) Representative image of mouse movement tracking of novel object recognition (NOR). (**I**,**J**) Recognition index for the mouse offspring. NOR groups used the same n as that in MWM. The data were evaluated as the means ± SEM. Statistical significance of MWM results was determined by a one-way ANOVA with a Dunnett’s multiple comparisons test. NOR results were analyzed using an unpaired two-tailed Student’s test. Data were analyzed per individual animal and not on a litter basis. # *p* < 0.05 vs. vehicle (**A**); * *p* < 0.05, ** *p* < 0.01 vehicle vs. TBBPA 0.24 mg/kg (**D**,**F**); ** *p* < 0.01, *** *p* < 0.001 increased recognition index for familiar object vs. novel object; ns, not significant (**I**,**J**).

The learning ability and memory of mice were evaluated using behavioral tests. The Morris water maze test was conducted to measure memory and spatial learning. In the acquisition phase, the mice were trained to learn the location of the platform by recognizing visual cues as evidence of its hidden location. During this phase, male offspring in the 2.4 mg/kg group exhibited abnormal spatial learning behavior, taking significantly longer to find the platform on day 7 (*p* = 0.0255) compared to the vehicle group (Figure 4A). On day 10, the test phase, the behaviors of the mice were appraised while the platform was removed. In this phase, male offspring exposed to 2.4 mg/kg of TBBPA exhibited a significantly lower frequency of passing through the platform area (*p* = 0.0262) and time spent there (*p* = 0.0027) than the vehicle group (Figure 4D,F). The other groups showed no significant differences (Figure 4A–G). Visual representative images displayed the movement path of a mouse from each group, and indicated that the 2.4 mg/kg TBBPA-treated mice were less aware of the platform location (Figure 4C). Considering the results observed in the acquisition and testing phases, perinatal exposure to TBBPA may cause spatial memory deterioration in male offspring. A novel object recognition test was then performed to evaluate the recognition memory. Figure 4H shows a representative schematic image. Ordinary mice tend to spend more time exploring new objects than familiar objects during the testing session [35]. As expected, the vehicle group spent more time exploring the novel object (*p* = 0.0047) (Figure 4I). In contrast, the males in the 0.24 mg/kg and 2.4 mg/kg TBBPA exposure groups did not show a preference for the novel objects (0.24 mg/kg: *p* = 0.3369; 2.4 mg/kg: *p* = 0.1350) (Figure 4I). All groups for female mice showed significant preference for the novel object (Vehicle: *p* = 0.0001; 0.24 mg/kg: *p* = 0.0002; 2.4 mg/kg: *p* = 0.0068) (Figure 4J). The results suggest that oral exposure to TBBPA can impair the recognition memory of mouse offspring, with a more pronounced effect in males.

#### 2.2.4. Administration of TBBPA Disrupted Social Interactive Behavior but Did Not Affect the Social Novelty

**Figure 5:**
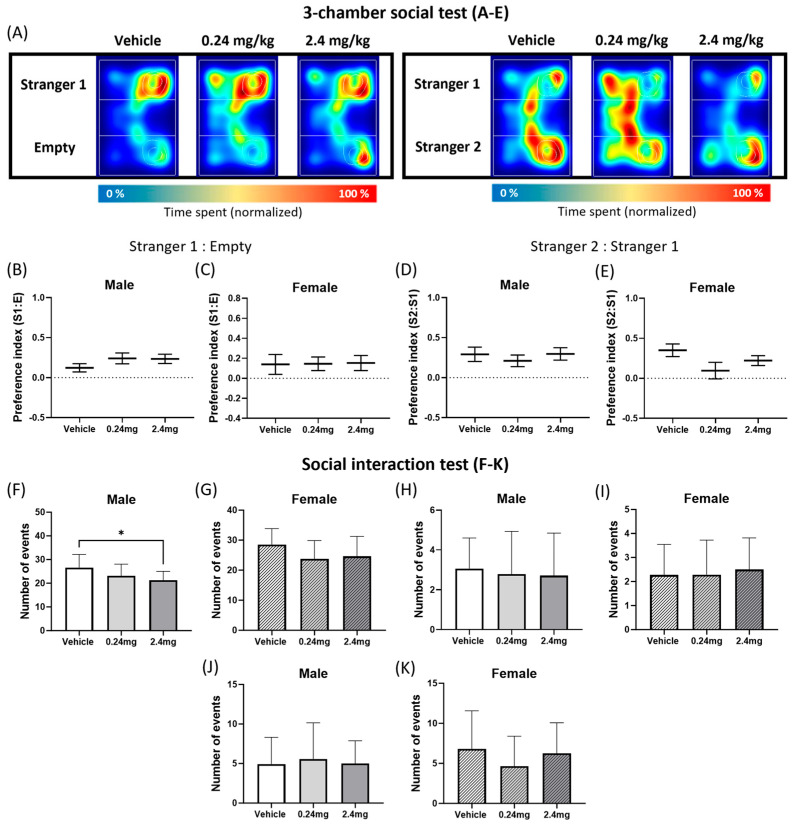
Effects of TBBPA on social interaction behaviors. (**A**–**E**) Three-chamber social test (3C) results. (**A**) Representative heatmap images of sociality and social novelty session. (**B**,**C**) Preference index of sociality session, *n* = 13 males/8 females for vehicle, 16 males/13 females for TBBPA 0.24 mg/kg, 7 males/13 females for TBBPA 2.4 mg/kg. (**D**,**E**) Preference index of social novelty session, *n* = 13 males/7 females for vehicle, 14 males/13 females for TBBPA 0.24 mg/kg, 2 males/13 females for TBBPA 2.4 mg/kg. (**F**–**K**) Social interaction test (SI) results: *n* = 15 males/11 females for vehicle, 19 males/16 females for TBBPA 0.24 mg/kg, 7 males/13 females for TBBPA 2.4 mg/kg. (**F**,**G**) The number of general sniffing events. (**H**,**I**) The number of anogenital sniffing events. (**J**,**K**) The number of following behavior. The data were evaluated as the means ± SEM. The statistical significance was calculated using a one-way ANOVA with a Dunnett’s multiple comparisons test. Data were analyzed per individual animal and not on a litter basis. * *p* < 0.05 vehicle vs. TBBPA 2.4 mg/kg.

The three-chamber social test was conducted to assess sociability-related behavior. Figure 5A shows a representative heatmap image. The results of the three-chamber test showed that TBBPA treatment does not influence social novelty (Figure 5B–E). In general, mice move closer to a stranger mouse than a familiar mouse [36]. In the experiment, all groups of mice showed a strong preference for unfamiliar mice (Stranger 1) over the empty cage. Therefore, all the preference indices were taken as positive values (Figure 5B,C). In the social novelty test, the Stranger 2 mouse was consistently preferred over the Stranger 1 mouse, and the preference indices were within the positive range (Figure 5D,E). There was a slight difference in the average preference index according to the group, but the differences among the groups were not significant. In addition, the numbers of general sniffing, anogenital sniffing, and following events were measured in the social interaction test (Figure 5F–K). A significant decrease in the general sniffing pattern was observed in the male 2.4 mg/kg group (*p* = 0.0457) (Figure 5F). These results suggest that exposure to TBBPA impaired certain aspects of sociability-related behaviors, as indicated by reduced general sniffing, but did not significantly affect social novelty.

#### 2.2.5. Effects of Perinatal Exposure to TBBPA on the Gene Expression of Behavior-Related Markers

1. Locomotor-related gene expression

**Figure 6:**
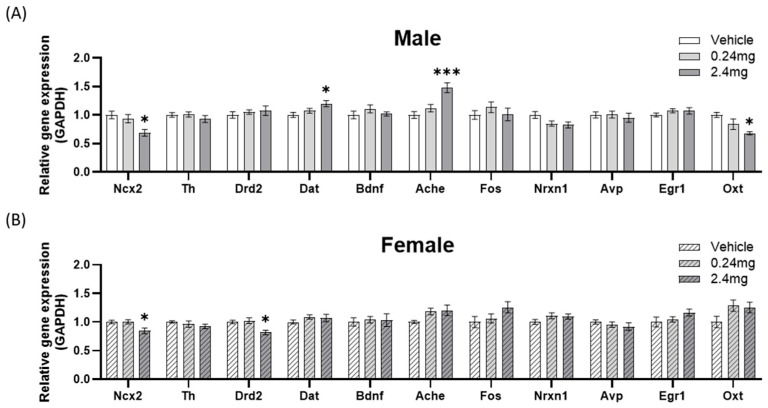
Changes in the expression of behavior-related genes in the brain. (**A**,**B**) Relative gene expression compared with *Gapdh*. *n* = 15 males/11 females for vehicle, 19 males/16 females for TBBPA 0.24 mg/kg, 7 males/13 females for TBBPA 2.4 mg/kg. The statistical significance was determined by one-way ANOVA with a Dunnett’s multiple comparisons test. Data were analyzed per individual animal and not on a litter basis. * *p* < 0.05, *** *p* < 0.001 vs. vehicle.

The expression levels of sodium-calcium exchanger 2 (*Ncx2*), tyrosine hydroxylase (*Th*), dopamine receptor D2 (*Drd2*), dopamine transporter (*Dat*) and brain-derived neurotrophic factor (*Bdnf*) were appraised as locomotor activity-related markers (Figure 6) [37,38,39]. The expression of *Ncx2* was lower than the vehicle group at 2.4 mg/kg in males (*p* = 0.0229) and females (*p* = 0.0233), but the *Th* and *Bdnf* levels were similar. *Drd2* expression was lower in the female 2.4 mg/kg group than the vehicle (*p* = 0.0222). *Dat* expression was higher in the male 2.4 mg/kg group than the vehicle (*p* = 0.0320). These gene expression changes may be associated with abnormal motor ability-related behaviors and have been induced by exposure to TBBPA.

2. Memory-related gene expression

The expression of several genes affecting memory was assessed. The previously mentioned *Bdnf* are also memory-related markers [40,41]. In addition, the expression of acetylcholinesterase (*Ache*), fos proto-oncogene (*Fos*), and neurexin 1 (*Nrxn1*) was evaluated as markers of cognitive and learning (Figure 6) [42,43,44]. Among the experiments on additional markers, *Ache* expression was higher in the male 2.4 mg/kg group than in the vehicle group (*p* = 0.0005).

3. Sociability-related gene expression

The expression patterns of the genes affecting social behavior were evaluated. *Fos* and *Nrxn1* also served as markers related to sociability [45,46]. The expression of vasopressin (*Avp*), early growth response protein 1 (*Egr1*), and oxytocin (*Oxt*) was evaluated as additional markers related to social ability (Figure 6) [47,48,49,50]. *Oxt* expression was lower in the male 2.4 mg/kg group than the control group (*p* = 0.0178), but there was no significant difference in the other genes.

## 3. Discussion

TBBPA is a brominated flame retardant widely used in plastics, electronics, and furniture. When disposed of, the compound leaks into the water, soil, and air. Humans are primarily exposed to TBBPA through food intake, and it has been detected in human blood and breast milk [51]. Some studies have reported that TBBPA should be considered safe because TBBPA is metabolized to apparently harmless metabolites [52,53]. Colnot et al. (2014) stated that TBBPA does not cause side effects that could be related to endocrine disorders [54]. On the other hand, many other studies have suggested that TBBPA may cause the endocrine disruption of the sex or thyroid hormones, and is therefore considered a type of EDC [23,25,55,56]. The brain is a target organ for the action of hormones secreted by the adrenal gland, thyroid gland, and gonads, which begin in the embryonic period when the hormone receptor sites appear on neurons [57]. During this period, estrogen exerts neurotropic, neuromodulatory, and neuroprotective effects that affect brain function, neuronal survival, and morphology. Therefore, estrogen can affect motor skills and mood, as well as memory and cognitive mechanisms [58]. Moreover, exposure to estrogenic EDCs during early development can influence brain development and function. Exposure of human females to EDCs is linked to early neurodevelopmental performance in offspring aged one to two years [59]. In mice, prenatal exposure to 4-tert-octylphenol impaired brain development and led to abnormal behavior [60]. These results suggest that early exposure to EDCs may cause neurodevelopmental impairment and induce abnormal behavior.

In this study, the developmental neurotoxic potential of TBBPA was predicted through a Sox1-GFP mESC-based screening assay. The Sox1-GFP cell line used in brain development-related research was based on GFP expression, which indicates the expression of the Sox1 gene, a gene related to central nervous system development [61]. The neurotoxicity and neurodevelopmental impairment results from Sox1-GFP cells were applied to the classification formula established in previous studies, confirming that TBBPA exhibits neurodevelopmental toxicity [33,34].

To verify the effects in a living organism, the effects of TBBPA on brain development and behavioral function in a sex-specific manner were evaluated by administering 0, 0.24, and 2.4 mg/kg/day of TBBPA orally to maternal mice, and performing behavioral experiments and gene expression pattern analysis using the resulting pups. The behavioral experiments revealed gender-specific effects. In females, only a deterioration of the motor ability was observed. In contrast, deteriorations in motor function, memory, and social interaction were noted in males.

In detail, there were no significant changes in the time spent in the center or the number of entries among all groups, both of which are indicators of anxiety in an open-field test. In the rotarod test, however, the latency to fall decreased in males treated with 0.24 and 2.4 mg/kg TBBPA and females treated with 2.4 mg/kg, suggesting some deterioration of motor ability. Similar results have been observed in studies using other EDCs. In a study using bifenthrin, a pyrethrin-based insecticide, rat offspring exposed to 54 mg/kg of bifenthrin during pregnancy and lactation did not affect anxiety in the open-field test but decreased the motor ability in the rotarod test significantly [62]. In another study, the pups of mice exposed to 0.3 mg/kg/day of perfluorooctane sulfonate during pregnancy also showed a decreased fall latency in the rotarod test for both males and females [63].

Beyond motor function, TBBPA also negatively affected spatial learning and memory. Male mice in the 2.4 mg/kg TBBPA group showed deterioration in the Morris water maze and novel object recognition tests. BPA, in particular, caused worsened spatial memory in male rats exposed perinatally to 2.5 mg/kg/day in the Morris water maze test, while prenatal exposure to 0.5, 5, and 50 mg/kg resulted in impaired object recognition memory compared to the control [64,65]. Prenatal alcohol exposure induced a deterioration of the reference memory in both male and female rats, but the working memory was impaired only in males [66].

TBBPA was also found to have a potential impact on social behavior. TBBPA did not affect social novelty, but it did cause a decrease in social interaction involving sniffing. As a similar precedent related to EDCs, diazinon at 0.18 mg/kg, in combination with perinatal exposure to 0.4, 1, and 2 mg/kg nicotine, caused no significant changes in the three-chamber test but decreased social interaction behavior in mice, while maternal exposure to di-(2-ethylhexyl) phthalate also impaired social interaction behavior in mouse offspring [67,68].

After behavioral assessment, gene expression analysis was conducted in the brain to identify gene expression changes associated with behavioral abnormalities. *Ncx2*, which regulates sodium and calcium ion exchange within neurons, was significantly decreased in the high-concentration group in male and female mice compared to the control group. Ncx2 plays a crucial role in maintaining calcium ion homeostasis, which is essential for neurons to generate the potential differences required for signal transmission [69]. If there is a problem with *Ncx2* expression, intracellular calcium ion accumulation occurs, leading to cell death and potentially causing various behavioral issues. In fact, its association with several neurodegenerative diseases has been reported [39,70].

*Dat* and *Drd2* expression changes were identified as being related to motor function. Changes in the expression of dopamine transporters and receptors involved in the dopamine signaling pathway are related directly to abnormal locomotor ability [38,71]. Dat is responsible for removing dopamine from the synapse and facilitating its reuptake. When *Dat* expression decreases, dopamine remains in the synapse for a longer duration, leading to increased signal transmission and hyperactivity [72]. Conversely, increased *Dat* expression results in rapid dopamine reuptake, reducing neurotransmission and potentially causing symptoms similar to those seen in Parkinson’s disease [72]. In this study, *Dat* was significantly increased in the high-concentration male group, which may be a contributing factor to the reduced motor performance observed in the motor assessment. The high-concentration female group, which exhibited reduced motor function, showed a decrease in *Drd2* expression. Drd2 is a G protein-coupled receptor that binds to dopamine and transmits signals, and its upregulation is associated with hyperactivity, while its downregulation can lead to motor impairments, as well as depression and attention deficits [73,74]. Despite the decrease in motor performance observed in the rotarod test for the low-concentration male group, no corresponding increase in *Dat* or *Drd2* expression was detected.

*Ache* expression increased in males of the high-concentration group. The Changes in AChE expression have also been reported in other rodent models of valproic-acid-induced autism [75]. Since acetylcholine is a crucial neurotransmitter for memory formation and learning, its increase can negatively affect memory, and several studies have reported that *Ache* is an important gene in cognition, depression, and memory [42,76]. The significant memory decline observed mainly in the high-concentration male group in this study and the significant increase in *Ache* expression exclusively in this group suggest a strong correlation.

Among the genes associated with sociability, changes in *Oxt* expression were also observed. Oxt regulates birth and lactation processes, and affects social behavior by regulating the spikes and synaptic plasticity in neural circuits [77]. In a previous study, social recognition behavior was disrupted in the male mice administered the Oxt antagonist [50]. A significant decrease in *Oxt* expression was observed exclusively in the high-concentration male group in this study, which also exhibited reduced sociability, suggesting a high likelihood that these behavioral changes were induced.

TBBPA exhibits low systemic bioavailability following oral exposure, and is rapidly metabolized into glucuronide and sulfate conjugates, primarily excreted via bile, with minimal accumulation in tissues [53,78]. Radiolabeled kinetic studies indicate that maternal TBBPA can cross the placenta and reach fetuses, though less than 1% of the administered dose was detected in fetal and placental tissues [79]. In contrast, lactational transfer via breast milk constitutes a more sustained postnatal exposure route, as demonstrated by detectable levels in pup stomach contents and livers, despite TBBPA’s overall low systemic bioavailability and rapid metabolism [4,79]. Moreover, environmentally relevant concentrations of TBBPA (1–100 nM) have been shown to modulate the activity of ATP-binding cassette transporters at the blood–brain barrier, based on studies using rat brain capillaries, including sex-dependent changes in P-glycoprotein and reductions in BCRP activity, while leaving MRP2 transport and barrier integrity unaffected [80]. Such transporter modulation, even without overt barrier disruption, may influence xenobiotic distribution and brain homeostasis during development. Together, these findings suggest that despite limited tissue accumulation, TBBPA’s ability to reach the developing brain and modulate neuroprotective transport mechanisms may underlie its potential to induce neurotoxic effects.

Overall, this study observed behavioral alterations and gene expression changes in offspring following maternal TBBPA exposure during pregnancy and lactation, though these effects were detected under experimental high-dose conditions that exceed typical human exposures. Notably, male offspring exhibited more pronounced effects, including impairments in memory, sociability, and motor function, whereas females primarily showed motor dysfunction, suggesting a relatively lower impact compared to males. These outcomes were largely confined to the high-dose group (2.4 mg/kg/day), which exceeds typical human exposures but was included to explore potential effects under experimental conditions.

The patterns of behavioral and genetic changes induced by TBBPA were consistently sex-specific, with males appearing to be more vulnerable than females. These sex-dependent effects, also observed with other EDCs such as microplastics, align with well-documented sex-based differences in brain development and behavior in both humans and rodents [81,82,83,84]. The sex differences in the effects of TBBPA may have multiple causes. First, sex hormones play a crucial role in mediating behavioral signaling through the brain. Changes in testosterone, progesterone, and estradiol levels can modulate contextual and spatial memory performance while also altering gene expression in the hippocampus, a region that regulates memory, cell morphology, and neurogenesis [85,86]. Second, sex differences in the gut microbiota–brain axis, including variations in microbial composition and responses to EDC exposure, have been shown to directly influence neurodevelopment and neural signaling [87]. Sex-specific alterations in the gut microbiota have been linked to many neurological diseases, including Alzheimer’s disease [88]. In this context, the neurodevelopmental and behavioral effects of TBBPA, an EDC with hormone-mimicking properties, are expected to exhibit sex-specific differences in responsiveness, highlighting the need for further research.

Although the selected doses are several orders of magnitude higher than current dietary exposure estimates—0.35 ng/kg/day for adults and 260 ng/kg/day for infants, as reported in EFSA’s 2024 reassessment [89]—they were chosen to ensure internal exposure and detect early hazard signals in exploratory screening. Furthermore, low oral bioavailability (<5%) in rodents justifies the use of such external doses [78].

While this study provides valuable preliminary insights into the neurodevelopmental effects of perinatal TBBPA exposure, several methodological limitations warrant discussion [90]. First, the study design does not fully conform to OECD Test Guideline 426 for developmental neurotoxicity (DNT) testing, particularly in terms of group size, exposure duration, and the use of litter as the statistical unit [91]. Litter-to-litter variation can substantially influence behavioral outcomes, accounting for up to 61% of total variance in some rodent studies [92]. Such effects can overshadow treatment-related differences and reduce the power to detect true neurobehavioral changes if not properly addressed. This highlights the need for caution in interpreting results based on individual-level analyses, as apparent statistical robustness may partly reflect unaccounted litter effects rather than genuine treatment responses. Due to the exploratory nature of the work and the limited number of available litters (e.g., *n* = 2 litters in the high-dose group), behavioral analyses were conducted at the individual level. While this deviates from regulatory standards, similar individual-level analyses have been employed in exploratory DNT studies to preserve statistical sensitivity and detect early biological signals [93,94,95]. In this context, it is important to consider that some GLP-compliant studies have reported no adverse neurobehavioral effects of TBBPA. For example, a two-generation reproductive toxicity study [96], as summarized in a review [97], reported no significant changes in motor activity, learning, or memory in rats exposed to TBBPA at doses up to 1000 mg/kg/day. However, this study was only available in abstract form, limiting the transparency and peer-review evaluation of its methodologies. Furthermore, while regulatory GLP studies ensure procedural consistency and reproducibility, they may not be sensitive to more subtle or sex-specific effects, particularly when behavioral assessments are limited to narrow endpoint categories. In contrast, our study utilized a broader behavioral battery encompassing emotional and cognitive dimensions, alongside sex-stratified gene expression analysis. While both studies applied high-dose exposures, our design aimed to elicit early mechanistic signals under realistic perinatal exposure timing. Therefore, rather than contradicting GLP-compliant studies, our findings serve to complement them by highlighting the need for expanded behavioral and molecular assessments when evaluating the neurodevelopmental risks of TBBPA.

Second, gene expression profiling was performed using whole-brain homogenates rather than region-specific dissections. Although this approach reduces anatomical resolution, it enabled the broad screening of molecular alterations potentially linked to behavioral changes.

Third, behavioral testing was conducted over a 10-week span (PNW6–16), raising the possibility that age-related factors may have interacted with treatment or sex effects. However, all groups were tested in a matched and counterbalanced order across the same developmental stages. Still, as neurobehavioral trajectories differ by age and sex, testing windows were selected to match key developmental stages in rodents that correspond to human vulnerability windows [98].

In conclusion, this study demonstrates that perinatal exposure to experimental doses of TBBPA induced neurodevelopmental alterations in offspring, accompanied by gene expression changes, with effects more pronounced in males. While the administered doses were substantially higher than typical human exposures, these results highlight the importance of investigating the potential neurodevelopmental mechanisms of TBBPA and identifying sensitive subpopulations that may be at risk.

## 4. Materials and Methods

### 4.1. Experimental Design

The developmental neurotoxic potential of TBBPA was predicted using a cell-based developmental neurotoxicity assay employing Sox1-GFP mESCs, and further evaluated in vivo through the oral administration of TBBPA to pregnant mice. To test the hypothesis that perinatal exposure to TBBPA impairs neurodevelopment in a sex-specific manner, offspring were evaluated in a series of behavioral tests, with primary outcomes defined as motor activity, memory performance, social interaction, and depression-related behaviors. Additionally, the expression of the genes associated with behavioral abnormality was investigated by quantitative PCR (qPCR) using brain tissue from the offspring.

### 4.2. Chemical

From the chemicals used, 3,3′,5,5′-TBBPA (97%) was purchased from Sigma-Aldrich (St. Louis, MO, USA).

### 4.3. mESC Culture and Neuronal Differentiation

The Sox1-GFP mESCs were obtained from Prof. Eekhoon Jho (Cellular Signal Transduction Lab, University of Seoul, Republic of Korea). The cells were cultured in 100 mm plates (Corning, Corning, NY, USA) coated with 0.2% gelatin in a CO_2_ incubator (5% CO_2_, 37 °C). The growth medium was comprised of Dulbecco’s modified eagle’s medium (DMEM; WELGENE, Gyeongsan, Gyeongbuk, Republic of Korea), 15% fetal bovine serum (FBS; Biowest, Rue de la Caille, Nuaille, France), penicillin (100 U/mL)/streptomycin (100 mg/mL; Biowest), L-glutamine (Gibco, Grand Island, NY, USA), 1-thioglycerol (Sigma-Aldrich), MEM Non-Essential Amino Acids (NEAA; Gibco), and mouse leukemia inhibitory factor (mLIF; Millipore, Burlington, MA, USA). The growth medium provided an undifferentiated growth condition for cells. The cells were maintained in a medium that was replaced daily, and subculture was performed once every two days. A differentiation medium consisting of DMEM/F12 (Gibco), penicillin/streptomycin, L-glutamine, 2-mercaptoethanol (Gibco), N2 supplement (Gibco), B27 supplement (Gibco), and bovine serum albumin (BSA) fraction V (7.5%) (Gibco) was used for neuronal differentiation.

### 4.4. Sox1-GFP-Based Developmental Neurotoxicity Screening Assay

Before the animal experiments, a Sox1-GFP mESC-based developmental neurotoxicity screening assay was conducted to evaluate the potential impact of TBBPA on neural development [33,34]. First, a cell counting kit-8 (CCK-8) assay was performed to determine the IC_50_ value, i.e., the half-maximal inhibitory concentration of TBBPA. A 70 µL aliquot of growth medium containing 7000 cells per well was added to a 96-well plate (Nunc by Thermo Fisher Scientific, Roskilde, Denmark). After 24 h, the medium was removed and replaced with 200 µL of growth medium containing various concentrations of TBBPA. After 48 h, the cells were rinsed with Dulbecco’s phosphate-buffered saline (DPBS; WELGENE) and incubated with a CCK-8 solution (Dogenbio, Seoul, Republic of Korea) for one hour. The absorbance of the plate was read at 450 nm using an Epoch spectrophotometer (BioTek Instruments, Inc., Winooski, VT, USA). The absorbance of the wells containing the control cells was set to 100% cell viability, and the absorbance of each well was compared. The IC_50_ value was calculated using GraphPad Prism 10 (GraphPad Prism Software, San Diego, CA, USA). A neural differentiation evaluation was conducted to obtain the ID_50_ value, i.e., the half-maximal inhibitory differentiation dose. One hundred cells were seeded in a 96-well round-bottom plate (Corning) with 140 µL of differentiation medium containing various concentrations of TBBPA per well. After four days, the area and the integral GFP of the formed neurospheres were measured using Lionheart FX (BioTek) and Gen5 Software (BioTek). The GFP intensity was calculated by dividing the integral GFP value by the area value, and the intensity of the control cell was set to 100% to obtain the ID_50_ value using GraphPad Prism 10. The discriminant function of the test method was as follows: 0.4345932 × logIC_50_ + 0.4295667 × logID_50_ + 2.687087; the compound was classified as toxic if the discriminant function score was less than zero.

### 4.5. Experimental Animals

Ten-week-old specific pathogen-free C57BL/6N male mice (25 ± 2 g) and female mice (20 ± 2 g) were obtained from Koatech (Pyeongtaek, Gyeonggi, Republic of Korea). All mice were reared in brown-colored polycarbonate cages (200 × 260 × 130 mm) under controlled environment conditions with the light cycle from 8:00 a.m. to 8:00 p.m., a relative humidity of 50 ± 10%, and a constant temperature of 23 ± 2 °C. Animal housing and breeding conditions were maintained as previously described [99]. After an adjustment period, female mice were mated at 12 weeks of age based on previous findings indicating that this age ensures high fecundity and consistent litter size [100]. Mating was performed overnight at a female-to-male ratio of 2:1. The day a vaginal plug was detected was marked as embryonic day (ED) 0, and the day the offspring was born as postnatal day (PND) 0. All the animal procedures were approved by the institutional animal care and use committee (IACUC) of Chungbuk national university (CBNUA-1962-22-02).

### 4.6. Chemical Treatments

In this study, based on previous rodent studies investigating the behavioral toxicity and pharmacokinetics of TBBPA [14,78,79], two doses (0.24 mg/kg/day and 2.4 mg/kg/day) were selected to represent a low-dose and a higher-dose group, intended to ensure measurable internal exposure in rodents given their low oral bioavailability, rather than to reflect typical human exposure levels. Female mice with confirmed or suspected vaginal plugs were randomly divided into three groups, and the group size (*n* = 5 per group) was selected based on previous studies [60,95]. Only female mice were treated daily with 0, 0.24, or 2.4 mg/kg TBBPA. After mating, the paternal mice received no further treatment and were euthanized. TBBPA was dissolved in corn oil (Sigma-Aldrich) and administered orally to the mice at 100 µL each through a 20-gauge oral gavage needle from ED10 to PND20. Among them, 3, 4, and 2 dams in the 0, 0.24, or 2.4 mg/kg groups, respectively successfully delivered pups. The litter size and sex distribution were recorded for each dam. In total, the vehicle group produced 26 offspring (15 males, 11 females), the 0.24 mg/kg group 35 offspring (19 males, 16 females), 2.4 mg/kg group 20 offspring (7 males, 13 females). The reduced number of litters in the 2.4 mg/kg group was attributed to unsuccessful pregnancies rather than maternal death or treatment-induced abortion. No maternal deaths were observed during the treatment period. After weaning (PND21), offspring were separated by sex and treatment group, and housed in groups of 3–5 and individually identified using ear notching. These offspring were used for subsequent analyses.

### 4.7. Behavioral Analysis

Behavioral testing was conducted from postnatal week 6 (PNW6), when mice are considered to have reached sexual and physiological maturity [101]. The mice offspring were selected arbitrarily, and all mice in each group participated in the full behavioral test series shown in Table 1. Test records were maintained to ensure that each mouse was used only once per test.

To minimize potential carry-over effects between tests, intervals ranging from two days to one week were given between two consecutive experiments. The behavioral test sequence was structured to proceed from less to more stress-inducing tasks while considering age-appropriate cognitive maturation and recovery needs [102]. Although the forced swimming test is recognized as a high-stress procedure, it was not positioned as the final assay in this study because the subsequent tasks—tail suspension test and Morris water maze test—are considered lower in acute stress and were spaced sufficiently to avoid cumulative stress effects.

Before each experiment, the mice were moved to the testing space and allowed to adapt for at least 30 min. The testing was conducted by laboratory technicians who were unaware of the mouse groups. All experimental areas were sanitized with 70% ethanol prior to and between each test. Individual mice were tested in isolation, without visual exposure to the behavior of other subjects.

#### 4.7.1. Open-Field Test

The open-field test was conducted in an opaque white chamber, 60 cm long, 60 cm wide, and 50 cm high. The mouse was placed gently in the front center of the wall facing one wall and allowed to explore the chamber freely for five minutes. The behavior of all mice was recorded and analyzed using EthoVision XT 14 software (Noldus Information Tech Inc., Leesburg, VA, USA). The time spent in the center (an imaginary square 30 cm long and 30 cm wide), the number of entries into the center, the distance traveled, and the speed were evaluated.

#### 4.7.2. Social Interaction Test

The social interaction test was performed in a cube-like acrylic arena with a length, width and height of 60 cm, 60 cm, and 50 cm, respectively, with a white floor. The mouse was placed in the front center of the wall facing one wall. A stranger mouse of the same sex was placed on the opposite wall. The two mice were allowed to move freely for 10 min, and the number of general sniffing, anogenital sniffing, and following were measured.

#### 4.7.3. Novel Object Recognition Test

The experiment was conducted inside a 60 cm long, 60 cm wide, and 50 cm high topless acrylic cube with a white bottom. The experiment consisted of two sessions: familiarization and testing. Each session was 10 min per mouse, and the interval between sessions was set to six hours. In the familiarization session, the mouse was placed in a cube containing two identical objects and explored freely. In the testing session, one old object and one new object were placed in a cube, and the mouse explored the cube. The mouse’s behavior was recorded, and the videos were analyzed using EthoVision XT 14 software. The time the mouse spent interacting with each object (smelling or exploring within 2 cm of the object) was assessed.

#### 4.7.4. Three-Chamber Social Test

The experimental apparatus comprised three plexiglass chambers arranged in a row. Each chamber was 20 cm wide, 40 cm long, and 22 cm high. The apparatus contained a small rectangular hole (10 × 5 cm) in the wall dividing the chambers, allowing the mice to move freely between each chamber. One side of both chambers contained a cylindrical cage (8 cm in diameter and 17 cm in height, with plastic bars spaced 1 cm apart). The test consisted of three sessions: habituation, sociability, and social novelty. In the habituation session, the test mice were allowed to explore the apparatus with empty cages freely for five minutes. For the sociability session, a strange mouse of the same breed, age, and sex (stranger 1 referred to as ‘S1’) was placed in a cage in one chamber, and the cage in the other chamber was left empty (empty, referred to as ‘E’). The test mouse was then placed in the middle chamber and allowed to explore all chambers freely for 10 min. For the social novelty session, another stranger mouse (stranger 2, referred to as ‘S2’) was placed in an empty cage, and the test mouse was then allowed to explore all chambers again for 10 min. Both sociability and social novelty sessions were recorded. The time spent in close proximity to the cage, distance traveled, and heatmaps were calculated using EthoVision XT 14 software. The preference index of each mouse was calculated using the following equation:

$$preference\;index=\frac{\left(S1-E\right)}{\left(S1+E\right)}or\;as\frac{\left(S2-S1\right)}{\left(S2+S1\right)}$$

‘E’, ‘S1’, and ‘S2’ represent the time spent close to the empty cage and stranger mouse 1 and 2 cages, respectively. Trials in which the subject completely climbed onto the top of the chamber walls were excluded from the analysis to avoid interference with standard behavioral parameters.

#### 4.7.5. Nest Building Test

The mice were placed one by one in a clean polycarbonate cage. At 19:00, each cotton nestlet measuring 5 × 5 cm and weighing an average of 2.5 g was given. After 12 h, the nest building ability was scored on a scale of 1 to 5 as follows: score 1 was given if the nestlet was noticeably untouched, with over 90% intact; score 2 indicated partially torn nestlet (50–90% remaining intact); score 3 was given if 50% or more of the nestlet was shredded but did not form a defined nest; score 4 was used when a flat but identifiable nest was formed, with over 90% shredded and collected in less than 25% of the cage floor and nest walls higher than the mouse body height in less than 50% of the perimeter; and score 5 was given for a well-structured, crater-shaped nest with over 90% torn nestlet and nest walls higher than mouse body height in more than 50% of the circumference [103].

#### 4.7.6. Rotarod Test

Mice were placed on a running rotarod (Panlab, Barcelona, Spain), and the time until they fell off the rod was measured (up to five minutes). The equipment was set to accelerate the rod from 5 to 40 rpm for five minutes. Each mouse was tested three times at 20 min intervals on days 1, 2, 3, 4, 6, 8, and 10. The protocol consisted of a training phase of three consecutive days, followed by a testing phase conducted every other day from days 4 to 10.

#### 4.7.7. Forced Swimming Test

A cylindrical glass tank (15 cm in diameter, 20 cm in height) was filled up to 12 cm high with tap water (25 ± 2 °C). Each mouse was gently placed in the tank and recorded for five minutes. The immobility time was measured using EthoVision XT 14.

#### 4.7.8. Tail Suspension Test

Each mouse was suspended by fixing its tail end 50 cm above the floor. The behavior of the subject mouse was recorded for six minutes. Videos were analyzed using EthoVision XT 14, and the duration of immobility during the last five minutes was assessed.

#### 4.7.9. Morris Water Maze Test

The experiment was conducted in a cylindrical metal tank, 90 cm in diameter and 40 cm in height, filled with tap water (25 ± 2 °C), made opaque by adding skim milk powder. The water surface was divided into four quadrants (I, II, III, and IV) based on the center point of the tank, and a circular platform was installed in the middle of the quadrant I (11 cm from the tank edge) at a depth of 5 mm from the surface. A visual cue was placed in the center of quadrant I on the inside face of the tank to serve as a spatial reference. Each mouse underwent an acquisition phase for nine consecutive days. At this phase, the mouse was placed gently facing the wall in the center of quadrants II, III, and IV (varied by the day of testing), and the escape latency and the time taken to reach the platform were measured. If any mouse did not reach the platform within 60 s, it was gently guided to the platform. The rest period on the platform was 30 s, and the subject was then placed back in the same position as before. During the acquisition phase, four trials were performed per day per mouse. On day 10 of the test phase, the platform was removed from the tank, and the mice were allowed to swim freely for one minute. Videos were recorded and analyzed using EthoVision XT 14. As indicators of spatial memory, the time spent in the location where the platform was and the number of times the location was passed were evaluated.

### 4.8. Anesthesia, Brain Tissue Collection, and cDNA Preparation

For anesthesia, Avertin (2,2,2-tribromoethanol: T48402; Sigma-Aldrich with tert-amyl alcohol: 240486; Sigma-Aldrich) was freshly prepared prior to use and applied as a single-use preparation. After behavioral testing was completed at PNW16, offspring mice were anesthetized via intraperitoneal injection followed by cervical dislocation, and whole brains were collected. The total RNA was extracted from the brain using TRI reagent (Invitrogen by Thermo Fisher Scientific, Waltham, MA, USA). An Epoch spectrophotometer was used to measure the total RNA concentration at 260 nm. The RNA quantified to 1 µg was synthesized into cDNA by reverse transcription using an iScript™ cDNA synthesis kit (Bio-Rad, Hercules, CA, USA), according to the manufacturer’s protocol.

### 4.9. Quantitative Real-Time PCR

Quantitative real-time PCR analysis was performed with QuantStudio 3 (Applied Biosystems, Foster City, CA, USA) using a 2× prime Q master mix and 50× ROX dye (GENETBIO, Daejeon, Republic of Korea), according to the manufacturer’s protocol. Table 2 lists the primer sequences. The amount of transcripts was quantified by a comparison with the internal control gene *Gapdh* based on delta Ct. The relative expression of each gene was calculated using the equation, R = 2^(−(∆Ct *target gene* − ∆Ct *Gapdh*)) and normalized relative to *Gapdh*. The expression in the vehicle group for each gene was set to one.

### 4.10. Statistical Analysis

The data were analyzed using a one-way analysis of the variance (ANOVA) with a Dunnett’s multiple-comparisons test or an unpaired two-tailed Student’s *t*-test. The overall statistical analysis was conducted using GraphPad Prism 10. Statistical details on behavioral assays are described in Supplementary Table S1.

## Figures and Tables

**Table 1:** Animal data for the behavior tests.

Test	Measurement	Number of Animals per Group	Age (Old)
Open field	Time in center (s) Number of entries into center (n) Distance moved (m) Velocity (cm/s)	15 male, 11 female for vehicle 19 male, 16 female for 0.24 mg/kg 7 male, 13 female for 2.4 mg/kg	6 weeks
Social interaction	General sniffing (n) Anogenital sniffing (n) Following (n) Fighting (n)	15 male, 11 female for vehicle 19 male, 16 female for 0.24 mg/kg 7 male, 13 female for 2.4 mg/kg	7 weeks
Novel object	Time around object (s)	15 male, 11 female for vehicle 19 male, 16 female for 0.24 mg/kg 7 male, 13 female for 2.4 mg/kg	8 weeks
3-chamber	Preference index	13 male, 8 female for vehicle 16 male, 13 female for 0.24 mg/kg 7 male, 13 female for 2.4 mg/kg	9 weeks
Nesting	Nesting score	15 male, 11 female for vehicle 19 male, 16 female for 0.24 mg/kg 7 male, 13 female for 2.4 mg/kg	10 weeks
Rotarod	Latency to fall (s)	15 male, 11 female for vehicle 19 male, 16 female for 0.24 mg/kg 7 male, 13 female for 2.4 mg/kg	11–12 weeks
Forced swimming	Immobile time (s)	15 male, 11 female for vehicle 19 male, 16 female for 0.24 mg/kg 7 male, 13 female for 2.4 mg/kg	13 weeks
Tail suspension	Immobile time (s)	15 male, 11 female for vehicle 19 male, 16 female for 0.24 mg/kg 7 male, 13 female for 2.4 mg/kg	14 weeks
Morris water maze	Escape latency (s) Distance moved (m) Velocity (cm/s) Platform crossing (n) Platform time (s)	15 male, 11 female for vehicle 19 male, 16 female for 0.24 mg/kg 7 male, 13 female for 2.4 mg/kg	15–16 weeks

**Table 2:** qPCR primer information.

Gene	Sequence (5′→3′)	
	Forward	Reverse
*Ncx2*	GTGGAATCATCATCGGGGCA	GGTCCTTATCCGGGTGCTTC
*Th*	TGCTCTTCTCCTTGAGGGGT	ACCTCGAAGCGCACAAAGTA
*Drd2*	AGTGAACAGGCGGAGAATGG	TAGACCGTGGTGGGATGGAT
*Dat*	ATGTGGTCGTGGTCAGCATT	CTGGCAGGCTGCAGAACTTA
*Bdnf*	GACAAGGCAACTTGGCCTAC	ATTGGGTAGTTCGGCATTGC
*Ache*	GCCTGAACCTGAAGCCCTTA	CTCGTCCAGAGTATCGGTGG
*Fos*	TACTACCATTCCCCAGCCGA	GCTGTCACCGTGGGGATAAA
*Nrxn1*	CCATCTGCATCTAGACCAGCC	TGCTGCTTTGAATGGGGTTTT
*Avp*	CGAGTGCCACGACGGTTTT	CAGCTGTACCAGCCTTAGCA
*Egr1*	TATGCTTGCCCTGTCGAGTC	GGATGTGGGTGGTAAGGTGG
*Oxt*	GAACTACCTGCCTTCGCCC	GAAGGAAGCGCGCTAAAGGT
*Gapdh* *(Internal control)*	TGCACCACCAACTGCTTAGC	GGCATGGACTGTGGTCATGAG

## Data Availability

The datasets generated during and/or analyzed during the current study are available from the corresponding author on reasonable request.

## Supplementary Materials

The following supporting information can be downloaded at: https://www.mdpi.com/article/10.3390/ijms26157289/s1.
