# Supplementary material for: Effects of TBBPA Exposure on Neurodevelopment and Behavior in Mice
Source: Int J Mol Sci. 2025 Jul 28;26(15):7289. doi: 10.3390/ijms26157289 (PMC12346716; doi:10.3390/ijms26157289)
Supplement: Supplementary file 1 [file ijms-26-07289-s001.zip › ijms-3732015-supplementary.pdf]

Supplementary Table S1. Behavior statistic data.

Test	Measurement		Mean ± SEM values	Statistical test		
				Test	Test statistic value	P value
Rotarod	Latency to fall (s)	Day1	M VE = 31.78 ± 2.95<br>M TBBPA 0.24 = 23.88 ± 2.22<br>M TBBPA 2.4 = 12.10 ± 2.22	One-way ANOVA with Dunnett correction test	$F_{2,120}$ = 9.648	0.0001
			F VE = 23.61 ± 3.80<br>F TBBPA 0.24 = 25.94 ± 2.98<br>F TBBPA 2.4 = 21.44 ± 3.13		$F_{2,117}$ = 0.5146	0.5991
		Day2	M VE = 56.67 ± 2.47<br>M TBBPA 0.24 = 40.37 ± 2.49<br>M TBBPA 2.4 = 19.05 ± 3.40		$F_{2,120}$ = 34.00	<0.0001
			F VE = 48.06 ± 4.94<br>F TBBPA 0.24 = 49.21 ± 3.33<br>F TBBPA 2.4 = 38.00 ±3.70		$F_{2,117}$ = 2.517	0.851
		Day3	M VE = 54.38 ± 2.54<br>M TBBPA 0.24 = 45.82 ± 2.59<br>M TBBPA 2.4 = 40.67 ± 5.06		$F_{2,120}$ = 4.309	0.0156
			F VE = 60.27 ± 4.69<br>F TBBPA 0.24 = 52.21 ± 3.81<br>F TBBPA 2.4 = 42.56 ± 3.32		$F_{2,117}$ = 4.594	0.0120
		Day4	M VE = 51.96 ± 2.67<br>M TBBPA 0.24 = 46.61 ± 3.03<br>M TBBPA 2.4 = 45.57 ± 4.81		$F_{2,120}$ = 1.037	0.3577
			F VE = 59.12 ± 4.90<br>F TBBPA 0.24 = 51.44 ± 3.37<br>F TBBPA 2.4 = 42.38 ± 3.05		$F_{2,117}$ = 4.577	0.0122
		Day6	M VE = 49.40 ± 2.77<br>M TBBPA 0.24 = 44.35 ± 3.32<br>M TBBPA 2.4 = 47.71 ± 6.33		$F_{2,120}$ = 0.5912	0.5553
			F VE = 66.21 ± 7.07<br>F TBBPA 0.24 = 59.50 ± 3.60<br>F TBBPA 2.4 = 50.62 ± 2.59		$F_{2,117}$ = 2.824	0.0.0634
		Day8	M VE = 52.89 ± 3.43<br>M TBBPA 0.24 = 43.77 ± 3.42<br>M TBBPA 2.4 = 41.62 ± 5.95		$F_{2,120}$ = 2.330	0.1016
			F VE = 66.09 ± 7.27<br>F TBBPA 0.24 = 59.77 ± 4.44<br>F TBBPA 2.4 = 54.49 ± 2.86		$F_{2,117}$ = 1.255	0.2890
		Day10	M VE = 46.16 ± 3.48<br>M TBBPA 0.24 = 36.42 ± 3.33<br>M TBBPA 2.4 = 40.10 ± 5.69		$F_{2,120}$ = 1.965	0.1446
			F VE = 61.30 ± 6.30<br>F TBBPA 0.24 = 55.63 ± 3.60<br>F TBBPA 2.4 = 44.41 ± 2.99		$F_{2,117}$ = 3.779	0.0257
Open field	Distance traveled (cm)		M VE = 2451.82 ± 92.82<br>M TBBPA 0.24 = 2564.30 ± 163.16<br>M TBBPA 2.4 = 2914.17 ± 176.79	One-way ANOVA with Dunnett correction test	$F_{2,38}$ = 0.2143	1.605
			F VE = 2647.31 ± 79.50<br>F TBBPA 0.24 = 2667.11 ± 83.48<br>F TBBPA 2.4 = 2425.99 ± 128.89		$F_{2,37}$ = 1.787	0.1816
	Velocity (cm/s)		M VE = 8.06 ± 0.33<br>M TBBPA 0.24 = 8.59 ± 0.56		$F_{2,38}$ = 2.167	0.1285

			M TBBPA 2.4 = 9.94 ± 0.66			
			F VE = 8.87 ± 0.27<br>F TBBPA 0.24 = 8.92 ± 0.28<br>F TBBPA 2.4 = 8.10 ± 0.44		$F_{2,37}$ = 1.828	0.1750
	Time in center (s)		M VE = 13.71 ± 2.26<br>M TBBPA 0.24 = 13.80 ± 1.49<br>M TBBPA 2.4 = 15.20 ± 2.08		$F_{2,38}$ = 0.1135	0.8930
			F VE = 10.56 ± 1.49<br>F TBBPA 0.24 = 12.11 ± 1.35<br>F TBBPA 2.4 = 13.06 ± 1.97		$F_{2,37}$ = 0.5403	0.5871
	Number of entries into center		M VE = 9.47 ± 1.33<br>M TBBPA 0.24 = 10.68 ± 1.15<br>M TBBPA 2.4 = 12.00 ± 1.31		$F_{2,38}$ = 0.6847	0.5103
			F VE = 9.09 ± 1.21<br>F TBBPA 0.24 = 8.81 ± 0.92<br>F TBBPA 2.4 = 8.08 ± 1.05		$F_{2,37}$ = 0.2348	0.7919
Nest building	Nesting score		M VE = 4.40 ± 0.13<br>M TBBPA 0.24 = 4.26 ± 0.13<br>M TBBPA 2.4 = 4.00 ± 0.22	One-way ANOVA with Dunnett correction test	$F_{2,38}$ = 1.287	0.2878
			F VE = 4.09 ± 0.25<br>F TBBPA 0.24 = 4.38 ± 0.13<br>F TBBPA 2.4 = 4.08 ± 0.14		$F_{2,37}$ = 1.114	0.3390
Forced swimming	Immobility time (s)		M VE = 211.41 ± 2.79<br>M TBBPA 0.24 = 218.30 ± 4.69<br>M TBBPA 2.4 = 219.20 ± 3.80	One-way ANOVA with Dunnett correction test	$F_{2,38}$ = 0.9496	0.3959
			F VE = 226.27 ± 2.85<br>F TBBPA 0.24 = 231.22 ± 4.30<br>F TBBPA 2.4 = 224.29 ± 3.53		$F_{2,37}$ = 0.9439	0.3983
Tail suspension	Immobility time (s)		M VE = 144.89 ± 7.76<br>M TBBPA 0.24 = 122.38 ± 9.40<br>M TBBPA 2.4 = 118.20 ± 8.96	One-way ANOVA with Dunnett correction test	$F_{2,38}$ = 2.221	0.1224
			F VE = 176.05 ± 16.81<br>F TBBPA 0.24 = 151.97 ± 13.39<br>F TBBPA 2.4 = 151.19 ± 10.87		$F_{2,37}$ = 0.9522	0.3951
Morris water maze	Escape latency (s)	Day1	M VE = 49.70 ± 2.31<br>M TBBPA 0.24 = 44.29 ± 2.33<br>M TBBPA 2.4 = 41.57 ± 3.44	One-way ANOVA with Dunnett correction test	$F_{2,161}$ = 2.169	0.1177
			F VE = 53.23 ± 2.30<br>F TBBPA 0.24 = 50.39 ± 2.25<br>F TBBPA 2.4 = 47.58 ± 2.52		$F_{2,157}$ = 1.267	0.2846
		Day2	M VE = 22.37 ± 2.66<br>M TBBPA 0.24 = 19.20 ± 2.01<br>M TBBPA 2.4 = 15.68 ± 2.63		$F_{2,161}$ = 1.358	0.2601
			F VE = 30.82 ± 3.60<br>F TBBPA 0.24 = 36.17 ± 2.96<br>F TBBPA 2.4 = 28.10 ± 3.21		$F_{2,157}$ = 2.033	0.1343
		Day3	M VE = 14.22 ± 2.34<br>M TBBPA 0.24 = 12.53 ± 1.88<br>M TBBPA 2.4 = 10.82 ± 2.59		$F_{2,161}$ = 0.4250	0.6545
			F VE = 22.50 ± 2.67<br>F TBBPA 0.24 = 19.53 ± 2.81<br>F TBBPA 2.4 = 16.29 ± 2.52		$F_{2,157}$ = 1.168	0.3138

		Day4	M VE = 9.42 ± 1.09<br>M TBBPA 0.24 = 13.71 ± 1.69<br>M TBBPA 2.4 = 10.68 ± 2.13		$F_{2,161}$ = 2.1797	0.1165
		Day4	F VE = 14.80 ± 2.50<br>F TBBPA 0.24 = 14.91 ± 2.40<br>F TBBPA 2.4 = 14.67 ± 2.21		$F_{2,157}$ = 0.002552	0.9975
		Day5	M VE = 8.00 ± 1.21<br>M TBBPA 0.24 = 11.66 ± 1.51<br>M TBBPA 2.4 = 12.04 ± 2.15		$F_{2,161}$ = 2.002	0.1384
		Day5	F VE = 15.16 ± 2.69<br>F TBBPA 0.24 = 16.86 ± 2.35<br>F TBBPA 2.4 = 15.27 ± 2.48		$F_{2,157}$ = 0.1556	0.8561
		Day6	M VE = 5.32 ± 0.56<br>M TBBPA 0.24 = 4.80 ± 0.54<br>M TBBPA 2.4 = 7.29 ± 1.58		$F_{2,161}$ = 2.202	0.1139
		Day6	F VE = 10.61 ± 1.91<br>F TBBPA 0.24 = 11.88 ± 2.00<br>F TBBPA 2.4 = 6.65 ± 0.78		$F_{2,157}$ = 2.696	0.0706
		Day7	M VE = 6.57 ± 0.73<br>M TBBPA 0.24 = 5.58 ± 0.48<br>M TBBPA 2.4 = 9.75 ± 1.53		$F_{2,161}$ = 5.779	0.0038
		Day7	F VE = 8.43 ± 1.20<br>F TBBPA 0.24 = 6.75 ± 0.95<br>F TBBPA 2.4 = 6.13 ± 0.87		$F_{2,157}$ = 1.248	0.2898
		Day8	M VE = 3.90 ± 0.58<br>M TBBPA 0.24 = 4.29 ± 0.34<br>M TBBPA 2.4 = 4.89 ± 0.72		$F_{2,161}$ = 0.6807	0.5077
		Day8	F VE = 3.64 ± 0.49<br>F TBBPA 0.24 = 5.17 ± 0.62<br>F TBBPA 2.4 = 5.48 ± 0.81		$F_{2,157}$ = 1.941	0.1470
		Day9	M VE = 4.27 ± 0.48<br>M TBBPA 0.24 = 4.66 ± 0.68<br>M TBBPA 2.4 = 6.36 ± 1.00		$F_{2,161}$ = 1.656	0.1941
		Day9	F VE = 6.50 ± 0.73<br>F TBBPA 0.24 = 9.59 ± 1.64<br>F TBBPA 2.4 = 5.35 ± 0.74		$F_{2,157}$ = 3.318	0.0388
	Platform crossing		M VE = 5.56 ± 0.49<br>M TBBPA 0.24 = 5.09 ± 0.51<br>M TBBPA 2.4 = 3.71 ± 0.54		$F_{2,120}$ = 2.040	0.1345
	Platform crossing		F VE = 4.91 ± 0.67<br>F TBBPA 0.24 = 5.19 ± 0.57<br>F TBBPA 2.4 = 5.90 ± 0.81		$F_{2,117}$ = 0.5221	0.5947
	Platform time (s)		M VE = 2.54 ± 0.26<br>M TBBPA 0.24 = 2.23 ± 0.21<br>M TBBPA 2.4 = 1.20 ± 0.18		$F_{2,120}$ = 5.457	0.0054
	Platform time (s)		F VE = 2.40 ± 0.40<br>F TBBPA 0.24 = 2.71 ± 0.28<br>F TBBPA 2.4 = 2.85 ± 0.39		$F_{2,117}$ = 0.3922	0.6765
Novel object recognition	Recognition index (%)		M VE: Familiar = 37.50 ± 5.76<br>M VE: Novel = 62.50 ± 5.76	Two−tailed Student's *t* test	*t* = 3.070	0.0047
			M TBBPA 0.24: Familiar = 46.63 ± 4.90<br>M TBBPA 0.24: Novel = 53.37 ± 4.90		*t* = 0.9733	0.3369
			M TBBPA 2.4: Familiar = 42.15 ± 6.93<br>M TBBPA 2.4: Novel = 57.85 ± 6.93		*t* = 1.603	0.1350
			F VE: Familiar = 38.68 ± 3.35		*t* = 4.783	0.0001

			F VE: Novel = 61.32 ± 3.35			
			F TBBPA 0.24: Familiar = 38.49 ± 3.83<br>F TBBPA 0.24: Novel = 61.51 ± 3.83		t = 4.244	0.0002
			F VE: Familiar = 37.74 ± 5.85<br>F VE: Novel = 62.26 ± 5.85		t = 2.964	0.0068
3-chamber	Preference index	Social ability	M VE = 0.12 ± 0.05<br>M TBBPA 0.24 = 0.24 ± 0.07<br>M TBBPA 2.4 = 0.23 ± 0.06	One-way ANOVA with Dunnett correction test	$F_{2,33}$ = 1.118	0.3389
			F VE = 0.14 ± 0.10<br>F TBBPA 0.24 = 0.15 ± 0.07<br>F TBBPA 2.4 = 0.15 ± 0.08		$F_{2,31}$ = 0.007559	0.9925
		Social novelty	M VE = 0.29 ± 0.09<br>M TBBPA 0.24 = 0.21 ± 0.07<br>M TBBPA 2.4 = 0.30 ± 0.13		$F_{2,26}$ = 0.2743	0.7623
			F VE = 0.35 ± 0.08<br>F TBBPA 0.24 = 0.10 ± 0.10<br>F TBBPA 2.4 = 0.22 ± 0.06		$F_{2,30}$ = 1.810	0.1811
Social interaction	Number of sniffing events		M VE = 26.60 ± 1.43<br>M TBBPA 0.24 = 23.11 ± 1.11<br>M TBBPA 2.4 = 21.29 ± 1.41	One-way ANOVA with Dunnett correction test	$F_{2,38}$ = 3.428	0.0428
			F VE = 28.55 ± 1.60<br>F TBBPA 0.24 = 23.81 ± 1.41<br>F TBBPA 2.4 = 24.62 ± 1.84		$F_{2,37}$ = 2.253	0.1193
	Number of anogenital events		M VE = 3.07 ± 0.40<br>M TBBPA 0.24 = 2.79 ± 0.49<br>M TBBPA 2.4 = 2.71 ± 0.81		$F_{2,38}$ = 0.1152	0.8915
			F VE = 2.27 ± 0.38<br>F TBBPA 0.24 = 2.31 ± 0.34<br>F TBBPA 2.4 = 2.54 ± 0.35		$F_{2,37}$ = 0.1545	0.8574
	Number of following events		M VE = 4.93 ± 0.88<br>M TBBPA 0.24 = 5.58 ± 1.05<br>M TBBPA 2.4 = 5.00 ± 1.09		$F_{2,38}$ = 0.1297	0.8788
			F VE = 6.82 ± 1.43<br>F TBBPA 0.24 = 4.63 ± 0.88<br>F TBBPA 2.4 = 6.23 ± 1.01		$F_{2,37}$ = 1.163	0.3238

M: Male; F: Female; VE: Vehicle; TBBPA 0.24: TBBPA 0.24 mg/kg/day; TBBPA 2.4: TBBPA 2.4 mg/kg/day.
